# Body Weight Control Is a Key Element of Motor Control for Toddlers’ Walking

**DOI:** 10.3389/fnetp.2022.844607

**Published:** 2022-03-24

**Authors:** Jennifer N. Kerkman, Coen S. Zandvoort, Andreas Daffertshofer, Nadia Dominici

**Affiliations:** Department of Human Movement Sciences, Faculty of Behavioural and Movement Sciences, Amsterdam Movement Science Institute (AMS) and Institute for Brain and Behaviour Amsterdam (iBBA), Vrije Universiteit Amsterdam, Amsterdam, Netherlands

**Keywords:** motor development, muscle synergies, muscle networks, gravity, toddlers

## Abstract

New-borns can step when supported for about 70–80% of their own body weight. Gravity-related sensorimotor information might be an important factor in developing the ability to walk independently. We explored how body weight support alters motor control in toddlers during the first independent steps and in toddlers with about half a year of walking experience. Sixteen different typically developing children were assessed during (un)supported walking on a running treadmill. Electromyography of 18–24 bilateral leg and back muscles and vertical ground reaction forces were recorded. Strides were grouped into four levels of body weight support ranging from no (<10%), low (10–35%), medium (35–55%), and high (55–95%) support. We constructed muscle synergies and muscle networks and assessed differences between levels of support and between groups. In both groups, muscle activities could be described by four synergies. As expected, the mean activity decreased with body weight support around foot strikes. The younger first-steps group showed changes in the temporal pattern of the synergies when supported for more than 35% of their body weight. In this group, the muscle network was dense with several interlimb connections. Apparently, the ability to process gravity-related information is not fully developed at the onset of independent walking causing motor control to be fairly disperse. Synergy-specific sensitivity for unloading implies distinct neural mechanisms underlying (the emergence of) these synergies.

## Introduction

Gravity greatly affects early motor development as body weight control is of great importance for human locomotion (Dietz and Duysens, 2000; Duysens et al, 2000). New-borns can generate coordinated alternations of the lower limbs (Thelen and Fisher, 1983; Thelen et al, 1987; Dominici et al., 2011) but are not able to walk unsupported, yet. Since they can step when they only have to support about 20–40% of their own body weight, the integration of loading-related information to oppose gravity and the ability to control the own body weight appears crucial.

Loading of the limbs influences the walking pattern. It arguably modulates the efferent output (Harkema et al., 1997) *via* the activation of Ib-afferents that appears in weight acceptance muscles (Finch et al, 1991), i.e., in extensors. In adults, it enhances the activity of (anti-gravity) muscles during stance and delays the initiation of the swing phase (Duysens and Pearson, 1980). When adults are unloaded, muscle activity and kinetics change, while kinematic and spatiotemporal parameters are merely affected (Ivanenko et al, 2002; Ivanenko et al., 2004; Apte et al, 2018). In contrast, toddlers show clear changes in their kinematic coordination during their first independent steps, when supported for more than 30% of their body weight (Dominici et al, 2007). This suggests that the reduction in gravity affects motor control during walking in toddlers differently than in adults (Ivanenko et al, 2007). Like in adults, in infants unloading may elongate the stance phase duration of walking (Pang and Yang, 2000) and infant stepping already renders adaptation to loading and other environmental changes (e.g., external perturbations, walking in different directions or at different speeds; Lam et al, 2003; Lam and Yang, 2000; Pang and Yang, 2000; Thelen and Cooke, 1987; Yang et al, 2005; Yang et al, 1998). Infants that are responsive to load changes tend to acquire functional motor skills at very young age (Vaal et al., 2000). This suggests the importance of early neurodevelopment to integrate load-related sensory information (Chang et al, 2006; Lacquaniti et al, 2012).

Stepping movements of infants are thought to emanate from embryonic interneurons in locomotor spinal circuitry (Forssberg, 1985; Lacquaniti et al., 2012). Inhibition of these spinal networks by descending cortico-spinal input appears mandatory to refine muscle activity (McGraw, 1940; Harkema et al., 1997; Grillner, 2011; Petersen et al., 2012; Vasudevan et al, 2016). Yet, whether this inhibition includes the sensorimotor integration of load-related information is unclear. If it does, one has to realise that neuromaturation is not completed at birth (McGraw, 1940; Berger et al, 1987; Vaughan et al, 2003), which may—in fact—explain why cortical control seems limited at a young age and the effect of unloading is different between toddlers and adults.

During the first year of life, motor behaviour develops gradually towards independent walking (McGraw, 1940). This development is accompanied by an increase in the number of locomotor muscle synergies from two to four, a number that persists in adults (Dominici et al., 2011). Locomotor muscle synergies are orchestrated patterns of co-activations in (groups of) muscles that are often considered essential for interlimb coordination, in particular, during walking. Here, we forward the hypothesis that the maturation of the cortico-spinal tract and, especially, that of afferent loading-related feedback are paramount for the emergence and control of the two supplementary locomotor synergies.

The contribution of neural circuits to motor control can be summarised as a network with a modular structure of neural structures and pathways. Network analysis has proven successful when mapping structural and functional connectivity in the brain (Sporns and Betzel, 2016), studying more general anatomy (Esteve-Altava et al, 2015; Molnar et al, 2017; Murphy et al., 2018; Powell et al., 2018) and unravelling physiologically interacting subsystems (Jeong et al, 2000; Bashan et al, 2012; Faes et al., 2014; Ivanov et al, 2016) or basic physiologic states (Liu et al, 2015; Ivanov et al, 2017; Rizzo et al, 2020). Casting such a diversity of subsystems in a network provides a comprehensive overview of many to many interactions (Bassett and Sporns, 2017; Balagué et al, 2020). Networks may “rewire” through changes in the task, coordination, or evolutional development (Lacquaniti et al, 2013) and, hence, can serve as an excellent means to identify corresponding changes in the (interactions of the) subsystems.

We altered body weight support (BWS) and explored its influence on motor output in toddlers at the onset of walking and in children about 6 months after their first independent steps. By this, we zoomed in on body weight control during the first experience of independent walking. We employed synergy analysis and determined the minimal number of locomotor muscle synergies. Expectedly, around the occurrence of the first independent steps, the two supplementary locomotor muscle synergies emerge and settle. We constructed functional networks with multi synergy-specific layers, so called muscle synergy networks (Kerkman et al, 2020). While traditional synergy analysis combines muscle groups by their co-activation, combining a set of synergies into a network provides direct insight into the interaction between them. As such, it allows for an encompassing study of functional changes in muscle activity during a transition in physiological coupling (Bashan et al, 2012; Ivanov et al., 2014).

We investigated the temporal activation patterns of locomotor muscle synergies as a function of BWS and complemented the analysis by muscle synergy networks to detail changes in spatial representation between groups. All children were likely to show a mature motor output that we expected to turn towards less mature temporal patterns in the presence of high BWS because of the unloading-induced lack of sensory feedback. This primarily applied to the younger, first-steps group. We predicted changes to mainly occur in the two just emerged (or still emerging) locomotor synergies (Dominici et al., 2011), in particular by their altered strength (or amplitude). We anticipated changes in muscle activation to be also visible in the spatial representation, or synergies’ weightings, given the known changes in co-contraction within and between the legs around the onset of independent walking (Yang et al., 1998). Accordingly, we expected that muscle synergies were accompanied by densely connected networks containing several (functional) clusters associated with the BWS level.

## Materials and Methods

### Participants

Sixteen typically developing children were included in this study (age range between 10.9 and 23.1 months, all born at term). Children were divided into two groups based on their walking experience, the FS and FS+ groups. In the FS group, we included toddlers during their first independent steps (within 3 weeks of walking experience) and in the FS+ group toddlers with around 6 months of walking experience. Seven children were measured two times (Supplementary Table A.1). Subjects visited the BabyGaitLab of the Department of Human Movement Sciences, Vrije Universiteit Amsterdam, wore a diaper during all measurements and walked without shoes. Familiarisation time was incorporated such that children had time to get comfortable to the lab and the experimenters. Ethical approval conform the Declaration of Helsinki was obtained at the Faculty of Behavioural and Movement Sciences (VCWE-2016–082) and parents signed the informed consent after a written and verbal explanation of the study.

To assess the first independent steps, we established regular contact with the parents that were monitoring their child’s walking ability. Recording sessions were scheduled as soon as the parents reported that the child was able to walk independently for at least four consecutive steps. This moment was defined as “walking onset” with which we determined the corresponding “walking age” (Supplementary Table A.1). We recorded the first unsupported steps in fourteen toddlers, (FS group, mean age 14.1, range (10.9–17.2) months old), and nine children were recorded about 6 months after the first independent steps (FS+ group, mean age 19.6, range (16.5–23.1) months old).

### Setup

The experimental procedure was adapted to the children such that one experimenter and one parent were located next the child to reduce the risks of falling and to make sure that the child always felt comfortable. Children were encouraged to make steps while supported by their hands or their trunk above a running treadmill. An additional weighting trial was recorded during each session while the child was standing or sitting quietly on the non-running treadmill for at least 2 seconds.

Treadmill speed was tuned to elicit stepping movements and adjusted to a comfortable speed for the child based on his/her walking capacity; mean walking speed 0.7 ± 0.3 and 1.0 ± 0.3 km/h for the FS and FS+ group, respectively. To assess the influence of body support on the motor output, we recorded trials while an experimenter firmly supported the child’s trunk with both hands while sitting on a bench behind the child and applied an approximately constant vertical force during several consecutive strides on the treadmill (Supplementary Figure A.1 A). In addition, the forearm of the experimenter holding the toddlers were supported to guarantee that an approximately constant vertical force was applied during consecutive strides and limit the possibility of imposing movements on the toddlers (Supplementary Figure A.1 B). Manual unloading was previously used in infants (Thelen and Cooke, 1987; Yang et al, 1998; Lam and Yang, 2000; Pang and Yang, 2000; Lam et al, 2003; Yang et al, 2005; Dominici et al., 2007; Vasudevan et al, 2016). It is a natural strategy adopted by parents to support their children during walking and avoids potential changes in the walking patterns through external equipment. The amount of body unloading was varied trial by trial to cover a wide range of levels from low until high level of BWS. Whenever feasible, additional trials were recorded while the experimenter held one or two hands or the trunk to stabilise the body during walking and supplied only limited vertical force, which was typically less than 20% of the body weight.

### Data Acquisition

Kinematic and video data were collected with a Vicon motion capture system (10 Vicon Vero v2.2 cameras and Vue Vicon camera, Oxford, UK) and sampled at 100 Hz. Vertical ground reaction forces were recorded with a force plate and sampled at 1 kHz (N-Mill 60 × 150 cm, Motek Medical BV, Amsterdam, the Netherlands). Force sensors with a sensitivity of 3N were installed in the force platform to measure low body weight values.

Electromyography (EMG) was recorded of 18–24 bilateral leg and back muscles. The following set of muscles was recorded simultaneously from both body sides: tibialis anterior (TA), gastrocnemius medialis (GM), gastrocnemius lateralis (GL), soleus (SOL), rectus femoris (RF), vastus medialis (VM), vastus lateralis (VL), biceps femoris (BF), semitendinosus (SEM), tensor fascia latae (TFL), gluteus maximus (GLM), erector spinae recorded at L2 (ES). The skin was cleaned with alcohol and micro golden Cometa golden disc-electrodes pairs (acquisition area of 4 mm^2^) were placed at the approximate location of the muscle with an inter-electrode distance of 10 mm. To minimise movement artefacts, pre-amplified EMG sensor units were attached with double tape to the skin of the child and fixed with elastic gauzes. EMG data were recorded using Cometa Mini Wave Wireless EMG system (Cometa srl, Italy) and sampled at 2 kHz after online band-pass filtering between 10 and 500 Hz. EMG, kinematic, force and video data were synchronised online.

### Data Analysis

#### Kinematics

We analysed the video recordings frame by frame to identify the foot strike and foot off events of both feet. A gait cycle was defined from the right leg starting with the foot strike (0%) to the consecutive strike of the right foot (100%). We considered a sequence of strides successful if at least three consecutive strides were present. The first and last stride in each sequence as well as jumps or other interruptions were excluded from subsequent analyses.

#### Body Weight Support

The vertical force data were low pass filtered (12th order bi-directional Butterworth filter, cut-off frequency at 20 Hz) and the average amount of force was computed per gait cycle. We specified the amount of external BWS as the percentage reduction of the mean vertical force during a stride compared to the estimated body weight that we determined during the weighting trial. In our previous work (Dominici et al., 2007), we showed significant differences in foot coordination when toddlers were supported for more than 30% of their body weight, while adults and older children showed only minimal changes. Based on these results, four levels of BWS were selected. Per subject, gait cycles were hence grouped into four different BWS levels: no (<10%), low (10–35%), medium (35–55%) and high (55–95%) support (Supplementary Table A.1).

#### Electromyography

EMG signals were visually inspected and pre-processed by linearly interpolating ±150 ms epochs around peaks that exceeded ten times the signal’s standard deviation. These data were high-pass filtered (30 Hz) with a second order bi-directional Butterworth filter and notch filtered (fourth order) to remove the power line artefact. Subsequently, EMG envelopes were extracted as modulus of the analytic signal (Myers et al., 2003; Boonstra and Breakspear, 2012). We applied a low-pass filter (second order Butterworth filter, cut-off frequency at 5 Hz) to obtain the slow-temporal changes in muscle activity. Finally, envelopes were time normalised to 200 samples per gait cycle (Ivanenko et al., 2005; Cappellini and Ivanenko, 2006; Dominici et al., 2011) and scaled to the mean amplitude per muscle of the low level of support (10–35% BWS). For every BWS level, we used the bilateral EMG patterns for all individual subjects and pooled all gait cycles of all subjects to determine the grand averages for both groups.

#### Muscle Synergies

Muscle synergies were estimated using non-negative matrix factorisation (NNMF, Lee and Seung, 1999) by a multiplicative update algorithm over one to seven synergies. Briefly, NNMF decomposes the original EMG matrix into (small number of) temporal patterns and weighting coefficients:

$$\text{EMG}=\overset{\text{n}}{\underset{\text{i}=1}{\sum }}{\text{P}}_{\text{i}}\cdot {\text{W}}_{\text{i}}+\text{error}$$

where P represents the temporal activation patterns of the synergies ( n×s matrix, where n denotes a pre-defined number of synergies, n≤m , where m is the number of muscles) and W the synergies’ weighting coefficients ( m×n matrix).

Per group, we estimated muscle synergies for the grand-averaged EMG activities of the four BWS levels and concatenated them to obtain a (s×k)×m matrix, where s=200 is the number of samples, m=24 the number of muscles and k=4 the levels of body weight support, yielding a 800×24 matrix. In this form, our NNMF resulted in temporal patterns per BWS level and fixed synergies’ weighting coefficients across the levels of support. We also decomposed the original EMG signals for all individual subjects, for which we used the averaged EMG activities per subject for every BWS level; the corresponding results can be found in Supplementary Figure A.2. The reconstruction quality of the synergies was determined as the contribution of the synergies to the Frobenius norm λ of the original signal ( EMG ):

$$\lambda^{\left(n\right)}=\left(1-\frac{∥EMG-W^{\left(n\right)}P^{\left(n\right)}{∥}_{F}^{2}}{∥EMG{∥}_{F}^{2}}\right)\times 100\%$$

The number of synergies was selected such that the reconstruction of the synergies should exceed 88% of the Frobenius norm of the original signal per level of support (Zandvoort et al, 2019; Kerkman et al., 2020; Bach et al., 2021).

For every resulting synergy, the mean amplitude of the temporal pattern was determined for every BWS level relative to the no-support level to assess changes in the amount of muscle activity across the gait cycle. We normalised the amplitude of the temporal pattern to the maximum value over the gait cycle to discard amplitude effects and to verify whether changes in the temporal pattern were induced by a change in the waveform itself. An enlarged normalised amplitude indicated a longer peak duration of the temporal pattern. Finally, to quantify the similarity between temporal patterns independent of amplitude, we estimated the circular cross correlation (Oppenheim et al., 2001) between different levels of support.

#### Muscle Synergy Networks

Network analysis (Bullmore and Sporns, 2009; Betzel and Bassett, 2017) was performed to compare the spatial representation of the muscle synergies between groups (Boonstra et al., 2015; Kerkman et al., 2018; Murphy et al., 2018). We constructed muscle networks (Kerkman et al., 2020) for which we first scaled the synergies’ weightings coefficients by the sum of the integrals of the temporal patterns to correct for overall amplitude effects. Using the outer product of the scaled synergies’ weightings, we obtained a 24×24 connectivity matrix per synergy. A bipartite muscle network (Murphy et al., 2018) was created, in which muscles served as nodes and where edges were given as the afore-defined weighted appearance of two muscles in the same synergy (i.e., the elements of the connectivity matrix). The connectivity matrices of all synergies were thresholded with an absolute threshold of 5·10^−5^ and we determined the density and the transitivity of every synergy network (Kerkman et al., 2020). Network density and transitivity served to quantify (changes of) network topology in terms of the number and clustering of connections, respectively (Rubinov and Sporns, 2010).

## Results

### Effect of Body Weight Support on Muscle Activity

**FIGURE 1:**
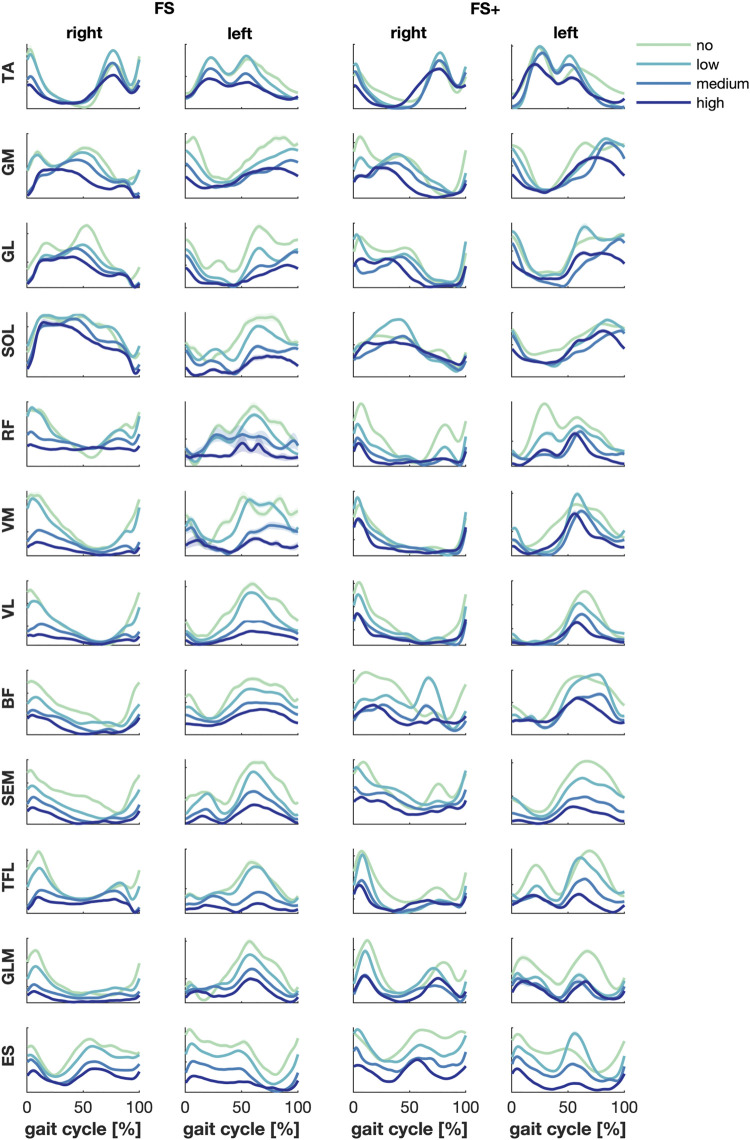
Muscle activities—grand average per group. Green, cyan, blue and dark blue represent no, low, medium, and high body weight support, respectively. Error patches represent the standard errors of the mean, which turned out very small.

The mean muscle activity over subjects per level of support showed a decreased amplitude and increased duration of the peak activity of several—mainly upper leg—muscles active at the foot strikes when weight support was increased (Figure 1). This effect was most pronounced in the FS group between low and medium BWS.

### Effect of Body Weight Support on Muscle Synergies

For both groups, four synergies were required to cover 88% or more of the original signal’s Frobenius norm (Table 1).

**TABLE 1:** Contribution of the synergies to the Frobenius norm ( λ ) of the original concatenated EMGs. λ was computed across all conditions as well as per level of support.

Group	# of synergies	Across conditions (%)	No (%)	Low (%)	Medium (%)	High (%)
FS	4	89	88	89	90	92
FS+	4	89	89	88	90	90

**FIGURE 2:**
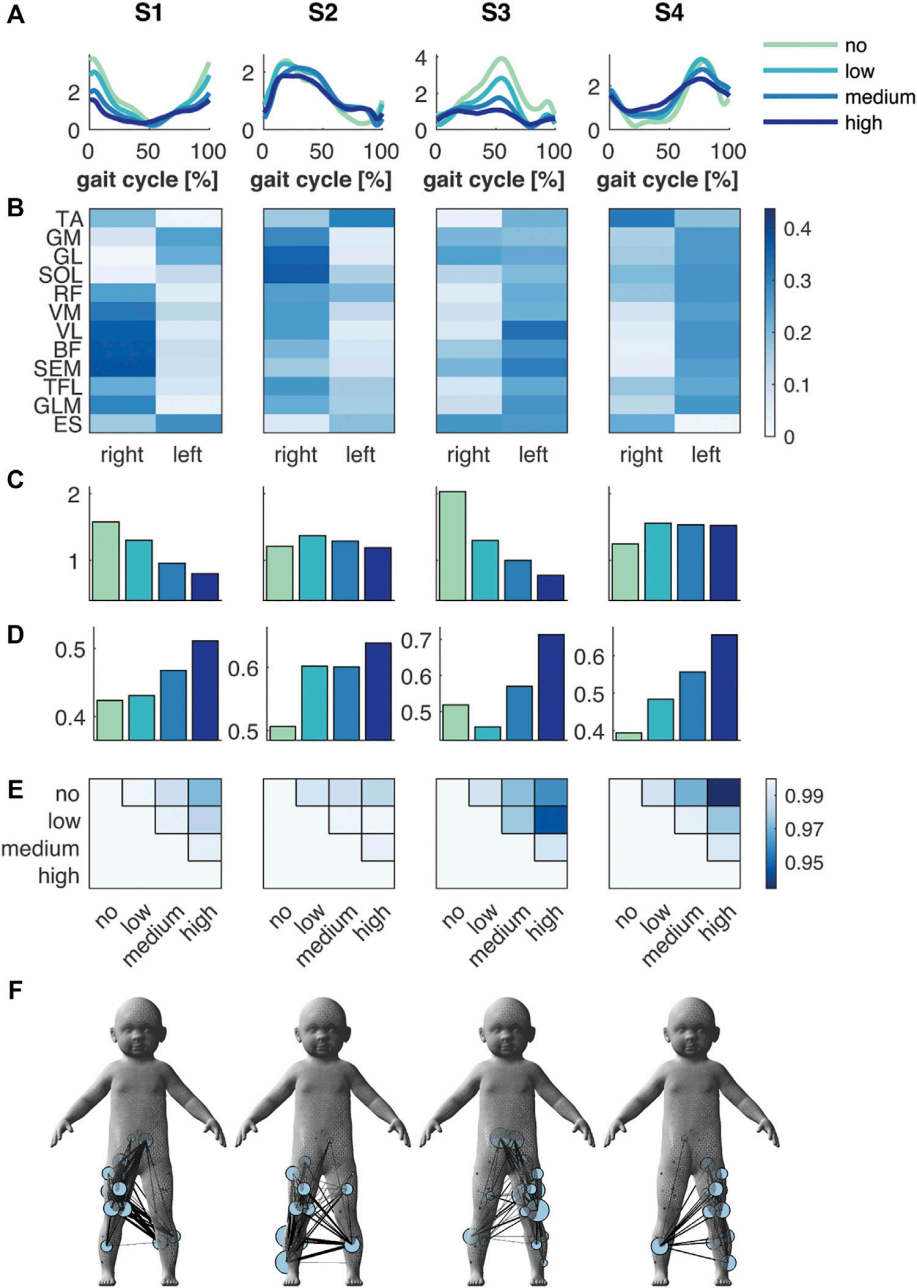
Muscle synergies and muscle synergy network in the FS group. **(A)** Temporal patterns and **(B)** synergies’ weighting coefficients. **(C)** Mean amplitude and **(D)** normalised mean amplitude of the synergy temporal pattern over the gait cycle, and **(E)** the circular cross correlation between the temporal pattern of the different levels of support. **(F)** Muscle synergy network on the toddler’s body mesh (MakeHuman 2018) based on the synergies’ weightings; node size represents the muscle degree and edge thickness the connection strength between muscles. Green, cyan, blue and dark blue represent no, low, medium, and high body weight support, respectively, in **(A,C,D)**.

Toddlers in the FS group displayed four synergies, of which two were primarily active during the right (S1) and left foot strike (S3), while the other two were active during the stance phase of the right (S2) and left leg (S4, Figure 2A). These right and left synergies appeared to be symmetric by mean of contributions of muscles of the right and left side (Figure 2B). The mean amplitude of the foot strike synergies decreased incrementally with respect to no support with unloading: −17, −39 and −49%, and −36, −51 and −62% for S1 and S3, respectively, whereas the mean amplitude in S2 mainly increased (+13, +6 and −2%) and in S4 remained almost constant between low, medium and high support (+25, +23 and +22%, respectively; Figure 2C). Next to the change in amplitude, the shape of the temporal patterns of S3 and S4 changed substantially: The circular cross correlation between no and high and low and high support decreased to 0.959 and 0.944 in S3, and in S4 between no and medium to 0.969 and between no and high support to 0.934 (Figure 2E). These changes seemingly reflected an elongated peak duration (Figure 2D).

**FIGURE 3:**
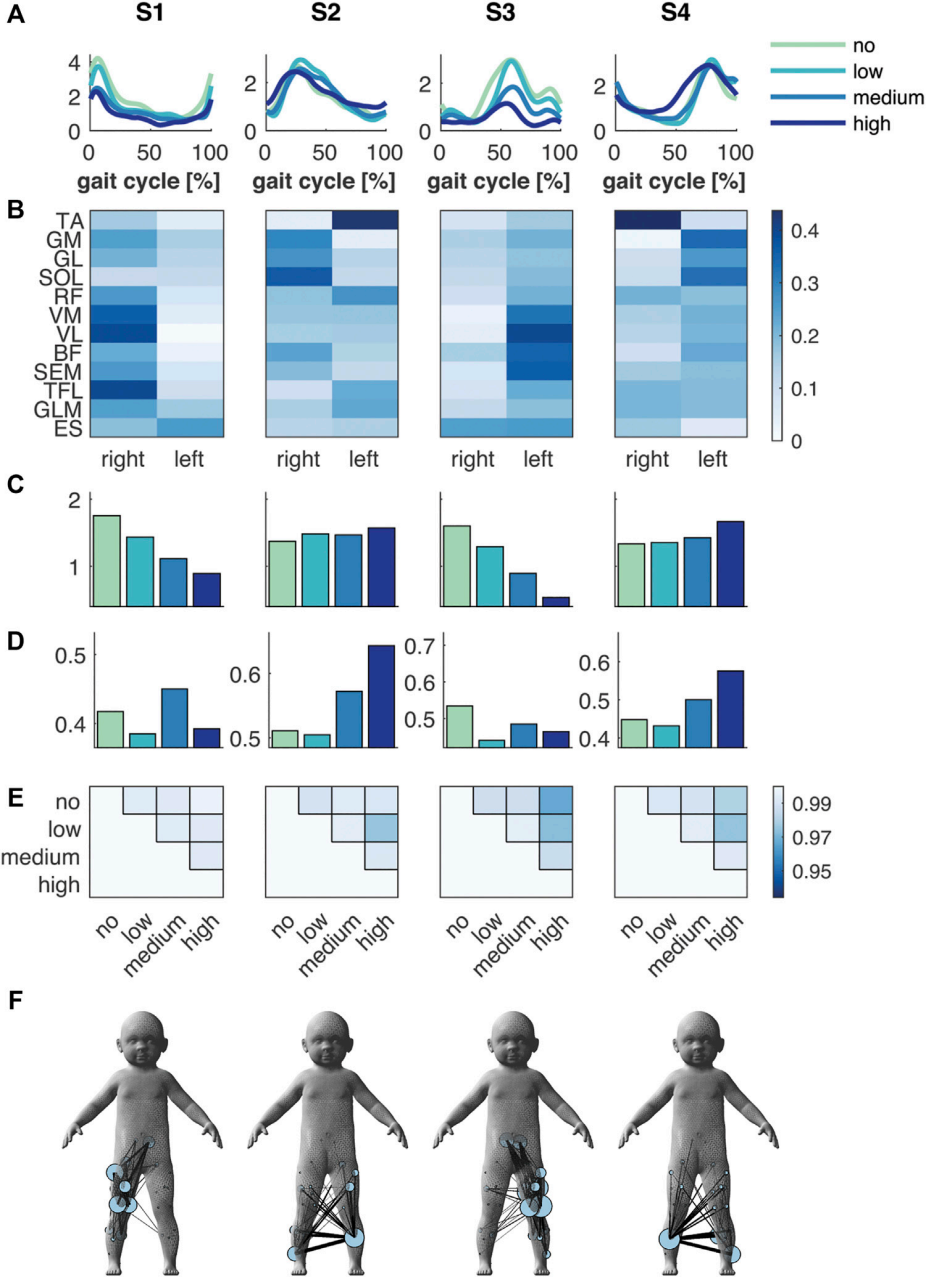
Muscle synergies and muscle synergy network in the FS+ group. **(A)** Temporal patterns and **(B)** synergies’ weighting coefficients. **(C)** Mean amplitude and **(D)** normalised mean amplitude of the synergy temporal pattern over the gait cycle, and **(E)** the circular cross correlation between the temporal pattern of the different levels of support. **(F)** Muscle synergy network on the toddler’s body mesh (MakeHuman 2018) based on the synergies’ weightings; node size represents the muscle degree and edge thickness the connection strength between muscles. Green, cyan, blue and dark blue represent no, low, medium, and high body weight support, respectively, in **(A,C,D)**.

The FS+ group also showed four synergies, which were like those in the FS group (Figure 3). Again, there was a foot strike synergy for both right and left leg (S1 and S3) and two synergies active during the right and left stance phase (S2 and S4). The mean amplitude of the temporal pattern decreased compared to the no BWS condition in S1 and S3 (−18, −37 and −49%, and −19, −44 and −67% in S1 and S3, respectively), while the mean amplitude of S2 and S4 increased (+8, +7 and +14%, and +2, +7, and +25%) in low, medium and high BWS, respectively. The similarity in shape of the temporal patterns (circular cross correlation) decreased to a minimum of 0.966 in S3 between no and high BWS, which implies that the temporal pattern barely changed in waveform in the FS+ group.

### Effect of Body Weight Support on Muscle Synergy Networks

The spatial representation of the muscle synergies of both groups revealed similarities in their muscle networks (Figures 2F and 3F). Yet, their network characteristics differed: the number of connections (density) of one muscle to another, i.e., whether muscles were active in the same synergy, was larger in FS compared to FS+ across synergies: +9, +70, +10 and +45%. Especially in the foot strike synergies (S1 and S3), we found a larger number of interlimb connections in the FS compared to the FS+ group. The transitivity was higher in FS compared to FS+ in all synergies except of S3 indicating more clusters in the synergy networks in toddlers at the onset of walking (cf. Table 2).

**TABLE 2:** Network density and transitivity per synergy for FS and FS+ Transitivity is $10^{-5}$ .

Group	Network metric	S1	S2	S3	S4
FS	density	0.21	0.23	0.28	0.21
FS+		0.19	0.13	0.15	0.15
FS	transitivity	7.9	5.2	4.6	5.2
FS+		5.8	3.8	5.5	2.8

## Discussion

For the currently study, we asserted the importance of body weight control in the development of independent walking. As expected, we found adaptations in temporal patterns of the muscle synergies when toddlers around their first independent steps were supported. We also found differences between the spatial representation between toddlers at the onset of walking and with half a year of walking experience. The motor pattern of these toddlers were similar to those of older children and adults as reported in the literature (Ivanenko et al., 2004; Ivanenko et al., 2005; Yokoyama et al, 2016). Both groups revealed four synergies with separate foot strike and stance phase synergies; the mean amplitude of the foot strike synergies decreased with increasing body weight support. It seems that, at the onset of walking, the coordination of muscle activity is reasonably developed allowing for independent walking, presuming a sufficient amount and quality of sensory feedback. However, the shape of the temporal pattern of the left foot strike and left stance phase synergies changed in toddlers at their first independent steps suggesting that the motor pattern in toddlers depends on the amount of support, while this dependency may be absent in older children, similar to adults (Ivanenko et al., 2004). Differences in spatial representation identified here demonstrated higher connectivity in the FS group compared to the FS+ group. This might have been caused by increased co-contractions and less specified contributions of muscles to the muscle synergies. The changes and differences arguably imply that the ability to control the body weight is a key element in the development of independent walking in children. In addition, the large number of interlimb connections found in the FS group are compatible with the idea that spinal network of interneurons project to multiple motor neurons pools, including distant motor neurons pools (Levine et al., 2014; Takei et al., 2017; Hug et al., 2021). These spinal networks seem to be largely involved in the coordination of toddler’s muscle activity during their first independent steps. Our results are in line with recent studies showing task-specific connectivity in the neuromuscular system during postural and walking tasks (Boonstra et al., 2015; Conway et al., 1995; Farmer et al., 1993; Kerkman et al., 2018; 2020).

Even when toddlers are only able to walk a few steps unsupported, four muscle synergies suffice to explain muscle activities during walking. This agrees with findings in older children and adults (Dominici et al., 2011). Here, the shapes of the temporal patterns were consistent across BWS levels. The amplitudes of the foot strike synergies, however, were clearly affected by BWS. This was probably due to a decrease in muscle effort to support the own body weight. It seems that the primary walking pattern is present at the onset of walking but that it can be modulated to account for body weight control requirements.

Despite the growing interest in the application of BWS in pediatric rehabilitation until now just few studies investigated the effect of body weight unloading in young children. We found changes in the muscle synergies of toddlers at the onset of independent walking in the shape of some of the temporal patterns when supported for more than 35%. This indicates that unloading affects motor control in these children. When unloaded, the available gravity-related information that can be transferred via Ib-afferents is reduced (Harkema et al., 1997; Pang and Yang, 2000). In toddlers at the onset of independent walking, the sensitivity and gain of the load-receptors might not be fully developed, and the motor system may not be able to account for a change in body weight support by modulating the gain of the feedback. The inability to integrate these load changes is supported by the observed overshoot of the foot in the swing phase in this age group (Dominici et al., 2007). When toddlers are unloaded for more than one third of their body weight, the information received by the neuromuscular system seems insufficient to preserve the primary motor pattern for walking, while this effect on motor control disappears in older children and adults when unloaded (Ivanenko et al., 2004).

BWS training has shown positive effects in the rehabilitation after stroke (Sale et al., 2012; Moraru and Onose, 2014) and spinal cord injury (Hubli and Dietz, 2013), and in the presence of Parkinson’s disease (Miyai et al., 2000; Picelli et al., 2013). In children with cerebral palsy, however, appear less promising (Damiano and DeJong 2009; Mutlu et al., 2009; Willoughby et al., 2009). Whether or not the diversity of finding in this degenerative disease has been cause by the age range of include patients remain opaque. Our results suggest that targeting load-regulating mechanisms in children should be most effective at very early age.

Striking is that only the newly developed synergies that are active during the foot strikes decreased in amplitude when unloaded. This suggests a phase-specific effect of body weight unloading, which has also been found in adults (Finch et al., 1991; Harkema et al., 1997; Sylos-Labini et al, 2014). The foot strike synergies may have a different origin than the stance phase synergies and they may be differently controlled. This supports a synergy-specific sensitivity for changes in the amount of body weight control. Some synergies may need proprioceptive feedback in the modulation of the synergy, while others operate without any proprioceptive or supra-spinal input (Grillner, 1973; Harkema et al., 1997) and, hence, remain largely unaltered despite of weight-bearing experiences during the first year (Yang et al, 2019). Cortico-muscular coherence found during the double support phase (Roeder et al., 2018) may suggest that the foot strike synergies are cortically controlled, while the other synergies may be controlled by brainstem and spinal networks (Labini et al., 2011; Lacquaniti et al., 2012). This arguably points at distinct neural origins of the synergies with different functions and sensitivities for gravity-related information.

By comparing toddlers at the onset of walking and half a year later, we found a changed spatial representation with a less densely connected muscle network in the younger group. Despite of similar muscle synergy activity patterns, the muscle clustering, and the contribution of muscles within the synergies evolved after a child started to walk independently. Yet, this reorganisation did not merely result from unloading (Supplementary Figure A.3), and changes in the networks were not consistent across synergies (e.g., no increased density in FS in all synergies). Hence, they could be the result of a combination of a decrease in co-contraction (Teulier et al, 2012) and a phase-specific reorganisation of muscle clustering.

### Limitations

The children involved in the current study were small, yielding limited space for EMG electrodes. Recall that we recorded multi-EMGs were recorded simultaneously. Potentially that may jeopardise data quality due to electrical crosstalk between adjacent muscles. However, the small size of the EMG electrodes used in our recordings and the chosen interelectrode distance should have minimised the pickup from adjacent muscles. Although it is not possible to separate co-activation from crosstalk in nearby muscles, muscle synergy analysis can identify whether a muscle is activated independent from a nearby muscle even in the presence of such crosstalk. Previous studies reported that if crosstalk did exist, it would likely have affected only the synergies’ weighting coefficients and not the number of muscle synergies or the temporal patterns (Ivanenko et al., 2004; Chvatal and Ting, 2013). Despite a proper skin preparation and EMG electrodes attachment, motion artefacts were still observed during foot strike in some of the lower limb muscles (TA, SOL). A pre-processing step was performed to the EMG signals to minimise these artefacts.

Muscle synergies are often estimated per subject (e.g., Ivanenko et al., 2004; Dominici et al., 2011). We must admit that in our toddlers’ group, it was quite difficult to collect EMG data from all muscles with sufficient steps in the four different BWS levels. To accommodate this, we averaged all steps across subjects. One may question the degree to which this grand average is representative for muscle synergies at single subject level. As expected, the single subject results were variable. Yet, when temporal patterns and synergies’ weightings coefficients were averaged over subjects, the results revealed similar temporal and spatial characteristics compared to the grand average results (Supplementary Figure A.2).

## Conclusion

Our results confirm that the ability for toddlers to control their own body weight is important in motor control during walking. Being at the onset of walking implies that the motor system can control independent walking. Yet, control processes continue to undergo modifications, arguably to integrate sensory feedback. Here, this was reflected in an amplitude decrease of the foot strike synergies when supported, i.e., a synergy-specific sensitivity of unloading. This can be a result of distinct neural mechanisms that may underlie the formation of synergies. Toddlers at the onset of walking showed a reorganisation of the spatial grouping of the muscles presumably due to immature motor control resulting in high co-contraction and less muscle-specific activity during the gait cycle. Unloading-induced motor adaptation was pronounced in these children when supported for more than 35% of their body weight. Apparently, motor control at the onset of walking is not fully developed, yet, and is modulated by loading-related feedback stressing its importance in the motor development of independent walking.

## Data Availability

The data that support the findings of this study are available on request from the corresponding authors.
