# Supplementary material for: Body Weight Control Is a Key Element of Motor Control for Toddlers’ Walking
Source: Front Netw Physiol. 2022 Mar 24;2:844607. doi: 10.3389/fnetp.2022.844607 (PMC10013000; doi:10.3389/fnetp.2022.844607)
Supplement: Supplementary file 1 [file DataSheet1.pdf]

Appendix

Subjects

Table A.1. Overview number of strides per subject and group.

Subject and session	age (months)	walking age (months)	weight (kg)	gender	no BWS	low BWS	medium BWS	high BWS
**FS**								
P1S1	12.1	0.2	8.7	female	56	101	37	78
P2S1	12.1	0.4	8.7	male	25	13	14	6
P3S1	17.2	0.5	9.4	male	77	213	33	30
P4S1	15.7	0.4	12.0	male	90	39	25	17
P5S1	17.1	0.4	11.7	female	7	70	13	3
P6S1	14.1	0.3	10.7	female	73	104	36	21
P7S1	13.1	0.2	10.3	female	19	189	72	38
P8S1	14.6	0.6	9.5	female	4	28	30	59
P9S1	10.9	0.3	10.7	male	0	89	92	60
P10S1	15.1	0.3	11.7	male	0	76	133	56
P11S1	14.8	0.6	11.2	male	0	116	109	94
P12S1	13.3	0.4	11.2	female	9	34	32	48
P13S1	13.9	0.2	11,8	female	13	250	111	25
P14S1	11.7	0.4	9.6	female	11	26	51	27
**Total**					**384**	**1348**	**788**	**562**
**FS+**								
P1S2	18.2	6.3	10.5	female	26	235	89	33
P4S2	20.8	5.5	14.0	male	92	146	110	40
P5S2	23.1	6.4	16.0	male	96	125	103	22
P6S2	19.4	4.5	11.7	female	85	37	53	38
P7S2	19.8	6.8	11.2	female	282	21	45	48
P8S2	20.1	6.2	11.0	female	141	22	1	4
P9S2	16.5	5.8	11.7	male	221	35	46	113
P15S1	19.5	5.9	11.1	female	3	28	6	23
P16S1	18.6	5.7	12.2	male	35	50	53	59
**Total**					**981**	**699**	**506**	**380**

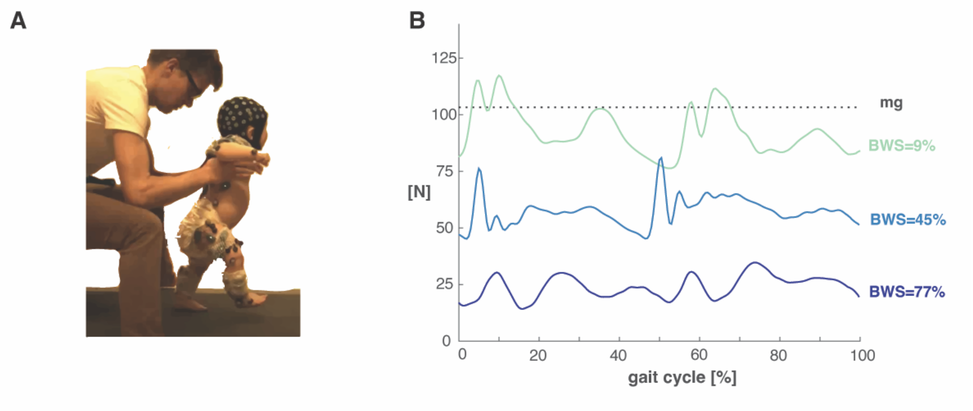

Figure A.1 Experimental setup. A) The experimenter firmly supported the child's trunk with both hands and applied an approximately constant vertical force during several consecutive strides on the treadmill and **B)** vertical ground reaction forces were recorded with a force plate under the treadmill. Dotted horizontal line indicate body weight. Amount of external body weight support was estimated as percent reduction of mean vertical force on the platform. Three examples of a same toddler (P1S2) walking with different amounts of BWS.

Muscle synergies per subject

Commonly, muscle synergies are estimated per subject (e.g. Dominici et al., 2011; Ivanenko et al., 2004). In our subject group, it was quite difficult to collect EMG data from all muscles with sufficient steps in the four different BWS levels. Therefore, we averaged all steps across subjects. Admittedly, it is questionable whether this grand average is representative for muscle synergies at single subject level. In fact, the single subject results were quite variable. However, when temporal patterns and synergies' weightings coefficients were averaged over subjects, the results revealed similar temporal and spatial characteristics compared to the grand average results (Figure A.2).

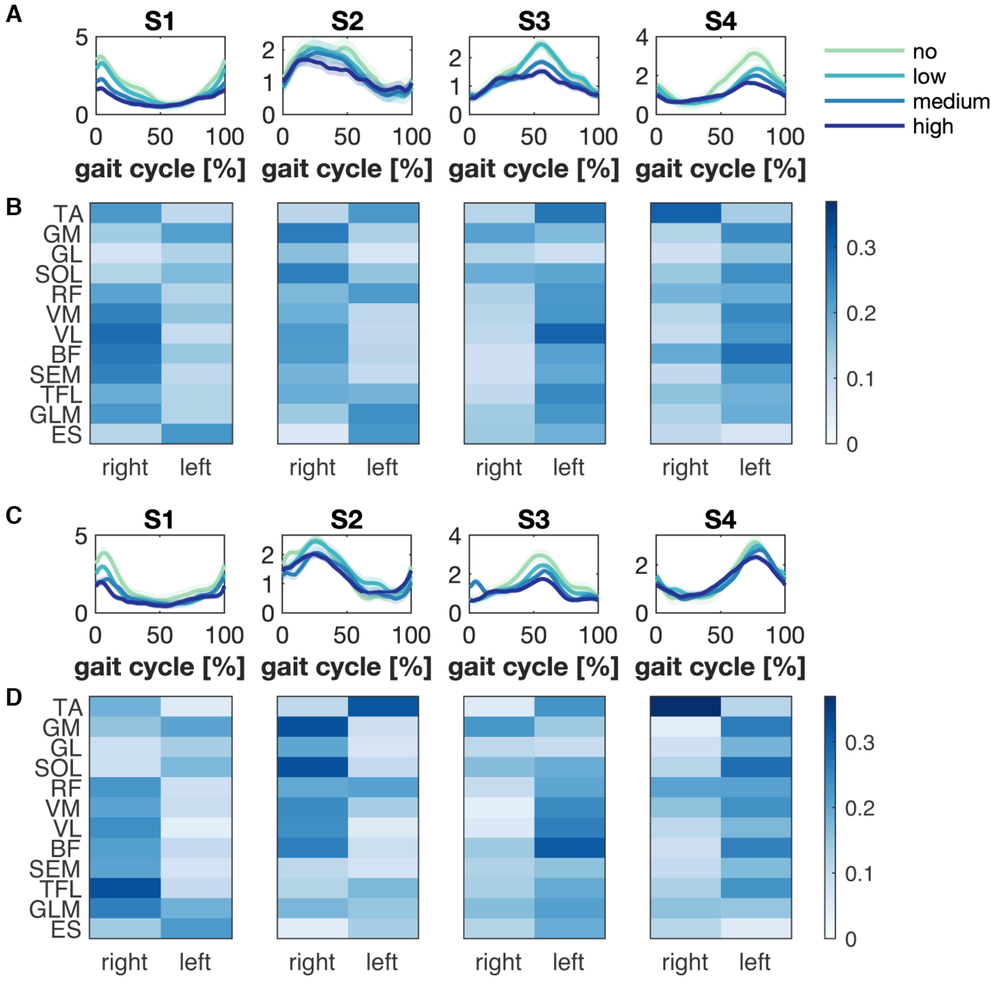

Figure A.2 Averaged single subject analysis. A) Averaged temporal patterns and **B)** synergies' weightings coefficients across subjects of the FS group, and **C)** averaged temporal patterns and **D)** synergies' weightings coefficients across subject of the FS+ group. Green, cyan, blue and dark blue represent no, low, medium, and high body weight support, respectively, in A and C. Shaded area represents standard error of the mean.

We consider this as a confirmation that the estimation of the muscle synergies of the grand average muscle activity was representative for the average muscle synergies.

Muscle synergy networks for unsupported walking

To evaluate whether the effect of the spatial reorganisation of the synergies is an effect of unloading, we estimated muscle synergies over the no support condition and created muscle synergy networks. The procedure was similar to the one described for the concatenated synergies, but instead of concatenating the averaged temporal patterns of all muscles, we estimated the synergies per temporal pattern of only the no support condition (<10% of the body weight). We used the synergies' weightings coefficient to construct muscle synergy networks. The connectivity matrices were thresholded with an absolute threshold of $2{\cdot}10^{-4}$ after which we determined densities and transitivities as summarized in Table A.2.

Table A.2 Network density and transitivity per synergy in the no support condition. Transitivity is $\cdot\, 10^{-4}$.

group	network metric	S1	S2	S3	S4
**FS**	density	0.11	0.22	0.09	0.21
**FS+**		0.21	0.28	0.09	0.14
**FS**	transitivity	1.6	2.3	1.0	2.4
**FS+**		2.1	2.1	1.4	1.4

The network density was higher in S1 and S2, remained constant in S3 and was lower in S4 in FS+ compared to FS in the no support condition. Transitivity was higher in FS compared to FS+ in S2 and S4 and lower in S1 and S3. This suggests that the spatial muscle synergy representation was altered between the groups of children in their number of connections between muscles, i.e. network density, and the clustering of muscles in the synergies, i.e. network transitivity, even though these children were barely unloaded. Hence, the reorganisation of the spatial representation of the muscle synergies was not merely an effect of unloading (see also Figure A.3).

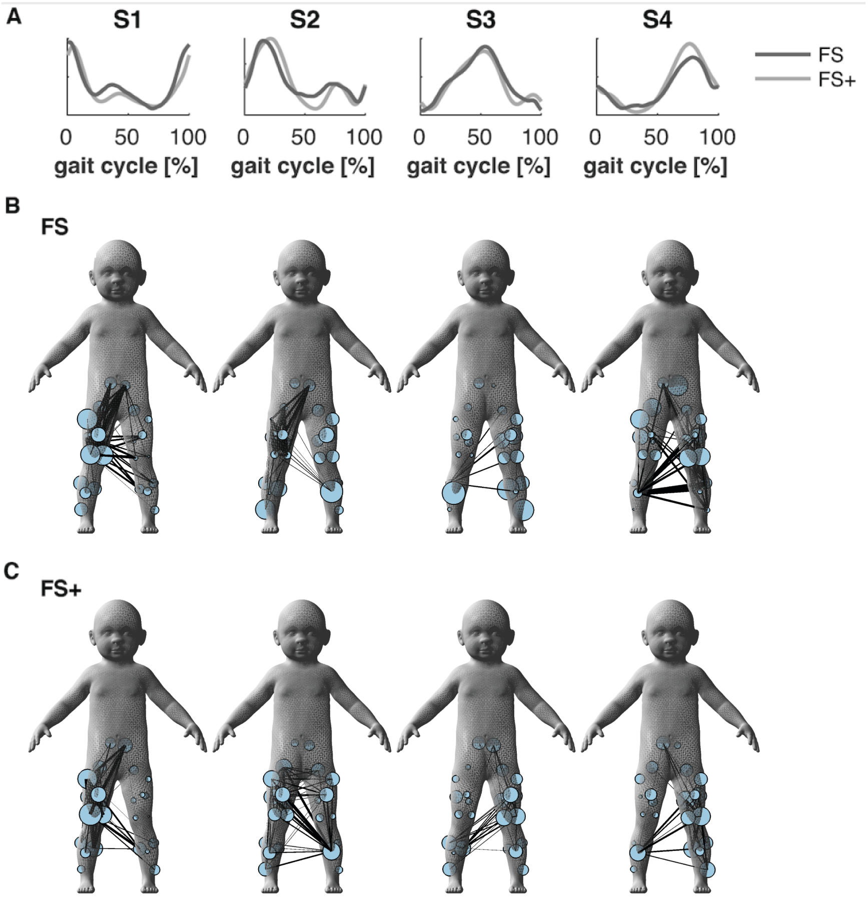

Figure A.3. Muscle networks for all synergies estimated for the no support condition. A) Temporal patterns and **B)** muscle networks of the FS group and **C)** muscle networks of the FS+ group. Node size represents the degree and the edge thickness the connection strength between two muscles.
